# Inversions and parallel evolution

**DOI:** 10.1098/rstb.2021.0203

**Published:** 2022-08-01

**Authors:** Anja M. Westram, Rui Faria, Kerstin Johannesson, Roger Butlin, Nick Barton

**Affiliations:** ^1^ Institute of Science and Technology Austria, Klosterneuburg, Austria; ^2^ Faculty of Biosciences and Aquaculture, Nord University, Bodø, Norway; ^3^ CIBIO, Centro de Investigação em Biodiversidade e Recursos Genéticos, InBIO, Laboratório Associado, Universidade do Porto, Vairão, Portugal; ^4^ BIOPOLIS Program in Genomics, Biodiversity and Land Planning, CIBIO, Campus de Vairão, 4485-661 Vairão, Portugal; ^5^ Ecology and Evolutionary Biology, School of Biosciences, University of Sheffield, Sheffield, UK; ^6^ Department of Marine Sciences, University of Gothenburg, Gothenburg, Sweden

**Keywords:** supergenes, local adaptation, parallel evolution, chromosomal rearrangements, gene flow

## Abstract

Local adaptation leads to differences between populations within a species. In many systems, similar environmental contrasts occur repeatedly, sometimes driving parallel phenotypic evolution. Understanding the genomic basis of local adaptation and parallel evolution is a major goal of evolutionary genomics. It is now known that by preventing the break-up of favourable combinations of alleles across multiple loci, genetic architectures that reduce recombination, like chromosomal inversions, can make an important contribution to local adaptation. However, little is known about whether inversions also contribute disproportionately to parallel evolution. Our aim here is to highlight this knowledge gap, to showcase existing studies, and to illustrate the differences between genomic architectures with and without inversions using simple models. We predict that by generating stronger effective selection, inversions can sometimes speed up the parallel adaptive process or enable parallel adaptation where it would be impossible otherwise, but this is highly dependent on the spatial setting. We highlight that further empirical work is needed, in particular to cover a broader taxonomic range and to understand the relative importance of inversions compared to genomic regions without inversions.

This article is part of the theme issue ‘Genomic architecture of supergenes: causes and evolutionary consequences’.

## 1. Background

Supergenes are genomic regions where alleles at multiple loci contributing to alternative phenotypes are kept in tight linkage [1,2]. In the most extreme scenario, a supergene acts as a single locus with a small number of discrete alleles, while with free recombination a variety of genotypes could be produced. This key feature of allowing a ‘switch’ between alternative genotypes without intermediates is useful where a set of optimal discrete phenotypes exists within a population, or where different discrete phenotypes are favoured in different populations connected by gene flow [3]. We here focus on the latter.

Supergenes might be generated by chromosomal inversions, which reverse the gene order in the affected chromosomal region [1]. A crucial consequence is that effective recombination between standard (ancestral) arrangements and inverted arrangements is reduced or largely prevented due to problems in meiosis. If each of the arrangements contains alleles contributing to local adaptation in a specific habitat, arrangement and habitat will become associated due to selection. Then, the inversion provides an efficient means of keeping adaptive allelic combinations intact and preventing the negative effects of recombination in areas of gene flow [4,5]. Inversions should thus be favoured where populations locally adapt to different habitats. Numerous empirical studies have shown that locally-adapted populations indeed frequently show strong differences in inversion arrangement frequencies [6], and in some cases specific locally-adapted phenotypes can be mapped to the inversion region [7–9].

**Figure 1.**
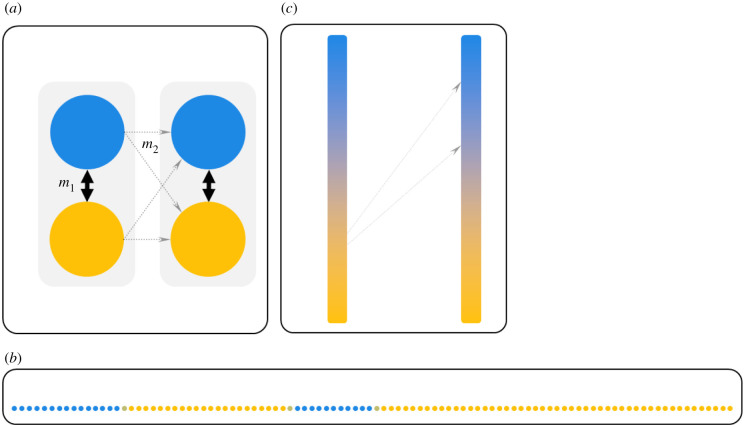
Example spatial scenarios of parallel evolution. In general, there must be at least two similar environmental transitions (here: from blue to yellow) in different locations; arrangement of demes, migration rates, and steepness of the environmental gradient can vary. Dispersal between neighbouring demes, or between nearby locations in a continuous gradient, is assumed to be high; long-distance dispersal is assumed to be rare (thin grey arrows). (*a*) Parallel evolution between two ‘islands’ each containing a similar environmental transition. We show the scenario modelled in §2, with high gene flow within islands (*m*_1_, thick arrows) and low, unidirectional gene flow between islands (*m*_2_, thin arrows). (*b*) Parallel evolution in continuous space (with migration between neighbouring demes). We show the scenario modelled in §3: to contribute to adaptation in the right blue patch (assumed to emerge later), blue alleles from the left blue patch must cross the yellow habitat, where they are deleterious. Light brown points represent edge demes with intermediate selection. (*c*) Parallel evolution between two smooth environmental gradients. Gradients are connected by rare, unidirectional long-range dispersal between locations not necessarily at the same position in the gradient.

In many species, local adaptation happens not just once: species often cover large and heterogeneous geographical ranges and experience similar environmental contrasts or gradients in multiple locations, leading to repeated divergence processes associated with the evolution of similar divergent phenotypes (parallel evolution) [10,11]. Schematic examples of repeated environmental contrasts are illustrated in figure 1 (we refer to different environments and the respective locally adaptive alleles as ‘blue’ and ‘yellow’ environments/alleles throughout this article). The question, then, is whether the genomic basis underlying adaptation is the same in these repeated instances of adaptation – i.e. whether local adaptation at the genomic level is repeatable [10,11]. Genomic studies can address this question. Results vary widely between systems, but in general, the similarity of the genetic basis appears to decline with geographical distance and time since divergence [12]. This may be explained by the fact that greater temporal and geographical proximity increases the chance that adaptive alleles are shared between locations, either via gene flow or via shared ancestral variation [12].

While both the number of studies on the genomic architectures of local adaptation (including the role of inversions) and the number of studies on parallel evolution are rapidly increasing, little is known about the intersection of these topics: How often do inversions contribute to parallel evolution? Are inversions more likely to drive repeated adaptation than loci in collinear regions of the genome (i.e. regions without inversion polymorphism)? With this article, we aim to highlight this knowledge gap and stimulate further research.

Why might inversions disproportionately contribute to parallel adaptation? First, because inversions can keep sets of locally adaptive alleles together in strong linkage disequilibrium, they might represent efficient ‘transport vehicles’ that can bring whole sets of adaptive alleles to a new location [13]. Second, because inversion polymorphisms might often experience stronger divergent selection than polymorphisms in collinear regions, they could be less likely to be lost by drift and thus more likely to be shared between different instances of local adaptation. Third, inversions may experience complex patterns of selection, including balancing selection [14], which can promote inversion polymorphism across large geographical scales and facilitate a contribution to parallel evolution. Fourth, because inversion content can evolve, the same arrangement might contribute to adaptation in different locations via different alleles at loci inside the arrangement, so it is not (only) the allelic content, but the inversion as a tool that contributes to parallel evolution.

On the other hand, alleles inside an inversion contributing to local adaptation will experience stronger negative selection whenever an arrangement is found in the ‘wrong’ environment, due to their strong association with other negatively selected alleles. This might impede the transport of arrangements across large geographical distances. In addition, because recombination between different arrangements is strongly reduced, inversions might be less flexible regarding their content and less efficient at removing deleterious variation than collinear architectures, which might also affect their role in parallel evolution.

To address the contribution of inversions to parallel evolution, studies must test whether parallel evolution has happened in a system, to what extent the genomic basis of adaptation is shared between locations, and to what extent inversions are involved and shared between locations. One reason that such studies are still rare is probably the fact that the detection of inversions and the determination of arrangement frequencies require specific analyses, which have not been performed for most systems. Even if inversions have been detected, the question of whether they contribute *disproportionately* to parallel evolution requires a statistical framework that is not fully developed (see ‘Avenues for future research’). In this article, we thus highlight empirical examples where a repeated role of inversions has been demonstrated, but stress that the relative role of inversions compared to loci in collinear regions is usually not known.

Our article has four main aims: 1. Highlighting empirical examples of a role of inversions in parallel evolution. Our main goal here is to show that inversions *can* contribute to parallel evolution, not to determine how common such a contribution is across study systems – as discussed above, for many systems it is currently impossible to distinguish the lack of a contribution of inversions from the lack of sufficient testing (but see [15,16] for counterexamples). 2. Illustrating conceptually why inversions might play a different role from collinear architectures, using simple simulations and theory. 3. Generalizing from the specific observations and models towards a list of factors that might favour or hinder the role of inversions in parallel evolution. 4. Encouraging future research.

## 2. Parallel evolution between ‘islands’

In this and the following two sections, we cover three different spatial scenarios (schematic examples in figure 1) with a review of empirical examples and simple models.

Adaptive divergence can happen repeatedly in different locations separated by unsuitable habitat and only connected by occasional long-distance migration (e.g. figure 1*a*). For example, a terrestrial species might inhabit similar habitat pairs on different islands.

### (a) Case studies

The stick insect *(Timema cristinae*) has a patchy distribution, with its habitat (host plant) occurring in an island-like fashion. Different colour morphs are cryptic on leaves and stems of host plants. An additional morph has a banding pattern providing camouflage on a host plant with needle-like leaves. Within each area of suitable habitat, different host plants occur in close proximity, either leading to mixing of morphs in full sympatry or small-scale migration-selection balance, thus fitting the model depicted in figure 1*a*. Genetic differences between morphs are concentrated in the *Mel-Stripe* locus, a large (10.5-Mbp) putative inversion [17]. Divergence between arrangements is probably evolutionarily relatively old (several million years; [18]) and arrangement polymorphism is present throughout the species range, locally maintained by balancing and divergent selection. While the *Mel-Stripe* locus is clearly associated with colour, it could not yet be confirmed that genes underlying phenotypic differences are located in this putative inversion [18].

Another example is adaptation on the between-island scale in the marine snail *Littorina saxatilis* (see §3 for within-island scale), which forms ecotypes adapted to steep cliffs exposed to wave action (Wave ecotype) and to habitats exposed to crab predation, e.g. boulder fields (Crab ecotype). Adjacent crab-inhabited and wave-exposed habitats occur in numerous coastal locations across Europe. Because there is no pelagic larva [19], parallel evolution matches an ‘island’ pattern requiring rare long-distance movement, e.g. via driftwood [20]. Multiple large (up to approximately 29.3 cM) genomic blocks, most of which likely correspond to chromosomal inversions [21], vary clinally in frequency across local cliff-boulder gradients [21,22] and show associations with divergent traits [9]. Importantly, these putative inversions differentiate ecotypes in many locations across Europe [23]. While arrangements could have risen to high frequencies from standing genetic variation present during initial colonisation, it is likely that they are sometimes transported between locations via long-distance gene flow.

Two ecotypes of the phenotypically variable Australian groundsel (*Senecio lautus* complex) are repeatedly adapted to sand dunes and rocky headlands following repeated colonization of coastal environments [24]. Loci underlying several locally adaptive traits cluster in a single genomic region [25], but it is not yet clear if the clustering is caused by an inversion.

In Atlantic cod (*Gadus morhua*) ancient chromosomal inversions are linked to life-history traits and local adaptation on both sides of the northern Atlantic [26–28]. NE and NW Atlantic populations diverged approximately 65 000 years ago; thereafter there were parallel splits into migratory and stationary ecotypes in several regions (e.g. Newfoundland, Iceland, Norway) [27]. Ecotype differences are strongly associated with the 0.6 million years old inversion on chromosome 1 on both sides of the Atlantic [27]. This supports a hypothesis of ecotype differences being carried around in an old inversion polymorphism and facilitating establishment of the two ecotypes in parallel in different regions of the species distribution.

### (b) Simulations

We modelled the ‘island’ scenario (figure 1*a*) to ask about key differences between inversions and collinear architectures regarding the extent and speed of parallel adaptation (see electronic supplementary material, information 1 for details). We are interested in the process of parallel evolution on island 2 after divergence on island 1 is already established. We focus on one specific, simple history, as we aim at illustrating what drives the differences between inversions and collinear architectures, rather than closely modelling a specific empirical system or covering a wide range of parameters or possible histories. After presenting illustrative simulations under a restricted parameter range we generalize using analytical theory.

We considered two islands each containing two demes (figure 1*a*). Migration between the two demes on the same island, *m*_1_, is much more frequent than long-range migration between islands, *m*_2_ (*m*_1_ = 0.01, *m*_2_ = 0.0001). Migration is unidirectional from island 1 to island 2, and the probability of arriving in either of the two demes is identical. We started with one yellow and one blue deme on island 1 and two yellow demes on island 2. When migration-selection equilibrium was reached (established adaptation on island 1), we changed one deme on island 2 to blue, and again ran to equilibrium to study parallel evolution.

There are two focal loci, each with a yellow and a blue allele. The blue allele is favoured in blue demes with selection coefficient + *s* and disfavoured in the yellow habitat with − *s*, and is present in the blue deme at the start of the simulation. There is no mutation, and all adaptation must be based on existing alleles. Individuals are diploid, selection is multiplicative within and between loci (no dominance or epistasis), and selection is soft (i.e. no change in population size).

We separately simulated the scenario with a collinear architecture (no inversion) and the scenario with an inversion. For the collinear case, there is free recombination between the two loci (and all possible two-locus genotypes can exist). For the inversion case, we assume that an inversion mutation has brought together the two blue alleles in the inverted arrangement, while the two yellow alleles are associated in the standard arrangement. Combinations of blue and yellow alleles in the standard arrangement could have existed in the past, but would have been removed by selection as they are not well-adapted in either habitat. There are thus only three possible genotypes (standard-standard, inverted-standard and inverted-inverted). Recombination is prevented in inverted-standard individuals, and the standard-standard and the inverted-inverted individuals are homozygous at both loci, so it is not necessary to model recombination.

**Figure 2.**
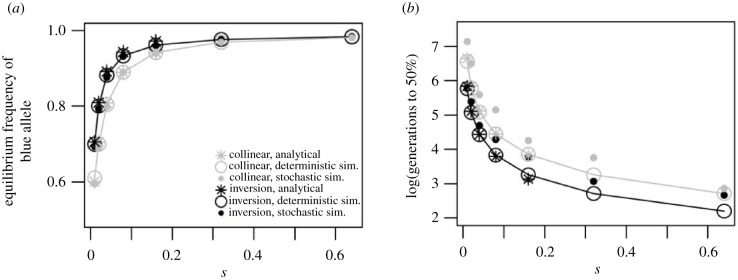
Parallel evolution on island 2 after established divergence on island 1 (in the scenario illustrated in figure 1*a*). (*a*) Equilibrium frequency of the blue allele in the blue deme on island 2, reflecting the extent of local adaptation. Results for an inversion (black) and a collinear architecture (grey) are compared. Large circles connected by a smoothed line represent results from deterministic simulations; Filled smaller circles represent average results of 10 replicate stochastic simulations with *N* = 500 individuals per deme; asterisks represent the analytical results (shown only for *s* ≤ 0.2 as the approximation is not valid for large *s*). (*b*) Time until the blue allele reaches half its equilibrium frequency in the blue deme on island 2 at both loci, indicating the time until parallel adaptation. Outcomes of individual stochastic runs can be found in electronic supplementary material, information 1, figure S1.

Compared to the collinear case, the inversion leads to a higher frequency of adaptive alleles in each deme on island 2 (figure 2*a*), and thus generates stronger parallel evolution between the two islands. This happens because in the inversion case, each blue allele experiences higher effective selection due to linkage with another blue allele. This effect is most pronounced when selection is weak, but is never substantial under the parameter values considered. Figure 2*b* shows that the time to parallel adaptation (here defined as the time to reach 50% frequency of the blue allele in the new blue deme) can be substantially reduced with an inversion compared to a collinear architecture, especially when selection is weak. This is because the inversion increases from low frequencies more rapidly once the environment changes, due to its higher effective selection coefficient. Comparing deterministic simulations (i.e. assuming infinite population sizes) and stochastic simulations (*N* = 500), we see that limited population sizes increase the time to adaptation, due to the stochastic loss of initially rare blue alleles in the new blue patch (figure 2*b*). However, adaptation is faster with an inversion than without in both the deterministic and the stochastic model.

### (c) Theory

To understand these simulation results more generally, we use analytical theory under the same basic assumptions.

Why can an inversion lead to better local adaptation (i.e. higher frequencies of the locally adaptive allele) than the collinear architecture in this model, and thus potentially contribute disproportionately to phenotypic parallelism? With a collinear architecture, the equilibrium frequency of a blue allele in the blue habitat on island 2, at strong selection relative to local migration (*m*_1_ ≪ *s*), is approximately 1 − (*m*_1_/*s*): there is some frequency of the maladapted yellow allele that decreases with the strength of selection (and is largely independent of the first island). With an inversion, the selection a blue allele experiences (*s*_e_) is roughly doubled because of perfect linkage to a second blue allele, and we need to substitute *s* by *s*_e_ ≈ 2*s*. Thus, with an inversion the frequency of the maladapted yellow allele is reduced, though not substantially (figure 2*a*). Under weak selection, the above approximation breaks down, but the frequency of the blue allele can be calculated numerically, and the difference between inversions and collinear architectures becomes even more pronounced (electronic supplementary material, information 2.1; figure 2*a*).

Why does parallel adaptation happen more rapidly with an inversion than with a collinear architecture? To understand this, we first consider the illustrative simpler case with just one deme per island, a blue deme fixed for the blue allele on island 1 and a blue deme that has just switched from yellow and initial fixation of the yellow allele on island 2. With a migration rate *m*_2_ into island 2, deterministically, the blue allele increases at a rate *m*_2_(1 − *p*) + *sp*(1 − *p*) and the time to go from *p* = 0 to *p* = 0.5 is log(2 + (*s*/*m*_2_))/(*s* + *m*_2_). For *m*_2_ ≪ *s*, this is ≈ (log(*s*/*m*_2_))/*s*, which only depends weakly on *m*_2_: even a very low rate of migration would lead to fixation in a time not much greater than 1/*s* generations.

In finite populations, establishment will take longer, because it takes a long time for an adaptive allele to arrive, and because not every arriving copy will establish. In each generation, 2*Nm*_2_ blue alleles arrive, and each has a probability 2*s* of being established. Therefore, they establish at a rate 4*Nm*_2_*s*, for *m*_2_ ≪ *s*, and the expected time to establishment is 1/(4*Nm*_2_*s*). The expected time to reach a frequency of 50% after arrival of a successful migrant is (1/*s*) log(4*Ns*). Overall, therefore, the expected time to reach 50% is 1/(4*Nm*_2_*s*) + (1/*s*) log(4*Ns*).

We can see that in this simpler scenario, in both the deterministic and stochastic model the time to adaptation decreases with *s* (figure 2*b*). Because *s*_e_ > *s*, adaptation is faster with an inversion.

We now consider the case of interest, with two demes per island, and with two initially yellow demes on island 2, one of which changes to blue. Half of the alleles that enter the newly blue deme via long-distance migration are blue (because the frequency of the blue allele on the first island *averaged across demes* is 0.5). The deterministic solution can be calculated numerically, and can be approximated in the limit of small *m*_2_ (electronic supplementary material, information 2.1). Analogously to the simpler scenario, the time to adaptation decreases with *s*, and is thus decreased with an inversion compared to a collinear architecture.

By the same argument as above, one can show that for a finite population, the time to reach 50% is

$$T=\frac{1}{2Nm_{2}\Pi }+\frac{1}{\lambda }\log(2N\Pi ),$$

where Π is the average establishment probability of the blue allele, and *λ* its rate of increase (defined in electronic supplementary material, information 2.1); this formula allows for the fact that only half the incoming alleles are blue, and that they enter both the blue and the yellow deme on the second island. Π is the average of the probabilities of fixation given that the allele lands in the yellow or the blue deme; these probabilities approach 0 and 2*s* when *m*_1_ ≪ *s* (i.e. Π ≈ *s*), whereas both are close to 2*λ* when *m*_1_ ≈ *s* (For derivation, see electronic supplementary material, information 2.1).

For low migration rates between islands, the time to increase after establishment is negligible (second term in the formula for *T*), and we can focus on 1/(2*Nm*_2_Π). If the migration rates within islands are also low (*m*_1_ ≪ *s*), *T* ≈ 1/(2*Nm*_2_*s*). With an inversion, *s*_e_ ≈ 2*s*, and thus the establishment time is simply roughly halved with an inversion compared to a collinear architecture. Importantly, this is the expected establishment time for a single locus. With an inversion, this is identical for the time for both loci because of perfect linkage. However, for the collinear architecture, the expected time until the adaptive allele at *both* loci establishes is approximately (3/2)*T*, leading to an additional advantage for the inversion (figure 2*b*). When *m*_1_ ≫ *s*, there is an additional speed-up with an inversion (electronic supplementary material, information 2.1).

## 3. Parallel evolution in continuous space

Parallel evolution can happen in continuous space (e.g. Figure 1*b*). By contrast to the first model, to contribute to parallel adaptation, a blue allele must now cross yellow habitat (where it is maladaptive) to reach and establish in a distant blue patch.

### (a) Case studies

In stickleback fish, freshwater adaptation from a marine ancestor has occurred repeatedly in different locations across the Atlantic and Pacific [29,30], and some genomic regions contribute to parallel adaptation across many locations [30]. The ‘transporter hypothesis' suggests that freshwater-adapted alleles entered the marine ecotype via introgressive hybridization from freshwater populations and were maintained over long periods in migration-selection balance [31,32]. They could thus be ‘transported’ across the marine environment and contribute repeatedly to freshwater adaptation.

Interestingly, in addition to loci in collinear regions, three inversions (*inv1*, i*nv11*, *inv21*) are involved in ecotype divergence [30]. They are relatively small (0.4–1.7 Mb), containing 21 to 75 genes [6,30]. They are likely present across the species range, highly divergent and relatively ancient (probably greater than 6 million years old [33]). However, QTL analysis revealed that only two known traits involved in ecotype divergence (armour plate number and body shape) are influenced by loci mapping to inversions, specifically to *inv21* [34]. A smaller fraction of the genome is involved in parallel divergence across oceans (Eastern Pacific and Atlantic) than within the Eastern Pacific, suggesting that the ability to cross an unfavourable habitat diminishes with distance [35]. Interestingly, among the reduced number of genomic regions involved in parallelism across oceans, three corresponded to the inversions [35].

Adaptation on the within-island scale in the marine snail *L. saxatilis* also reflects adaptation in continuous space. Along the shores of Swedish islands, boulder fields and cliffs alternate, and spread of locally adaptive alleles is much more likely via small-scale crawling rather than via the open sea. Therefore, a cliff-adapted allele must cross a boulder field to contribute to adaptation on the next cliff, and vice versa. Inversions strongly contribute to repeated adaptation also at this scale [36]. The inversions, though divergent in frequency between ecotypes, are often not near fixation within ecotypes [36], suggesting some degree of balancing selection. This would facilitate transport of adaptive arrangements between different cliffs or different boulder fields.

Another example can be conjectured for altitude adaptation in East African honeybees (*Apis mellifera*) [37]. Multiple pairs of neighbouring highland and lowland populations show repeated phenotypic divergence, and a phylogenetic tree is consistent with parallel divergence. A comparison between three such pairs, separated from each other by approximately 200–350 km, revealed two shared highly divergent genomic regions corresponding to previously suggested inversions (sizes: 0.573 and 1.639 Mb; ages: 3.2 and 1.8 million years [6]) [37,38]. Although the exact historical scenario is unclear, it is reasonable to speculate that the inversions involved in adaptation had a single origin and are now shared across highland populations due to ‘transport’ across lowland populations.

The cases discussed here suggest that inversion arrangements have travelled through habitats where they are maladaptive (figure 1*b*). However, alternative historical scenarios can usually not fully be ruled out, even for well-studied cases like stickleback freshwater-marine divergence [35,39]. In addition, the different studies suggest that the contribution of inversions to parallel evolution is likely to differ among systems.

### (b) Simulations

We modelled the situation where local adaptation is established in a single patch in continuous space (left blue patch in figure 1*b*) and parallel evolution occurs in a second, newly established patch (right blue patch in figure 1*b*).

We consider a one-dimensional chain of demes connected by roughly Gaussian dispersal (with standard deviation 1.5; see electronic supplementary material, information 1.2 for details). Selection and recombination were simulated as described above.

We simulated 100 demes, starting with an initial blue patch (first 15 demes) and the remaining demes consisting of yellow habitat. We ran the simulations until migration-selection equilibrium, resulting in local adaptation to the initial blue patch and limited flow of blue alleles into the adjacent yellow habitat. To simulate the emergence of a new blue patch in which parallel evolution could happen, we then changed the selection coefficient in a second set of adjacent demes (12 demes centred at deme 45), and again ran until equilibrium.

**Figure 3.**
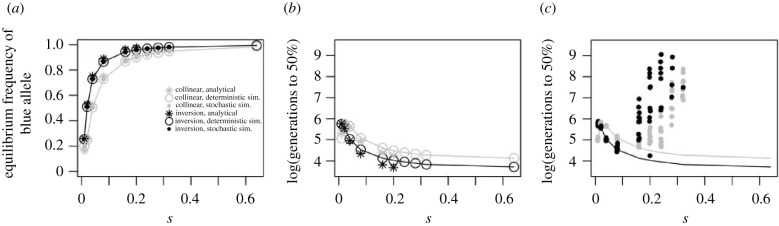
Parallel evolution in a second patch in continuous space after established adaptation in a first patch (in the scenario illustrated in figure 1*b*). We show the equilibrium frequency and establishment time for the blue allele in the centre of the new patch of blue habitat, comparing an inversion (black) and a collinear architecture (grey). (*a*) Equilibrium frequency. Large open circles, connected by a smoothed line, represent results from deterministic simulations; filled circles represent average results of 10 stochastic simulations at *N* = 10 000 individuals per deme; asterisks represent the analytical results (shown only for *s* ≤ 0.2 as the approximation is not valid for large *s*). For *s* = 0.64, the blue allele never increased in frequency within the 10 000 generations of stochastic simulation (see *c*), and thus no equilibrium frequencies could be determined (filled circles missing). (*b*) Deterministic simulations and analytical prediction for establishment time (time until the blue allele reaches 50% of its equilibrium frequency at both loci; symbols as in (*a*)), reflecting the time until parallel adaptation. (*c*) Establishment time in stochastic simulations. The deterministic result from (*b*) is shown again for comparison (lines). 10 replicate simulations were run for each value of *s*, but because the blue allele did not always establish within the 10 000 generations of the simulations, the number of points varies (and there was never establishment for *s* = 0.64).

Under most selection coefficients, the inversion enables a higher equilibrium frequency of the adaptive allele (figure 3*a*), and thus more pronounced parallel adaptation, compared to the collinear scenario. The benefit of the inversion is strongest when selection is relatively weak and the patch is vulnerable to maladaptive gene flow. In particular, at low values of *s* parallel adaptation might be impossible without an inversion because selection acting on each locus individually is not strong enough to prevent swamping by yellow alleles.

In deterministic simulations, the establishment time of the blue allele, and thus the time until parallel adaptation, is clearly decreased with an inversion (figure 3*b*). This suggests that the lower starting frequency in the inversion case (due to stronger counter-selection while the patch was still yellow) is outbalanced by a faster frequency increase under positive selection.

However, in stochastic simulations, the establishment time is dramatically increased, particularly at high values of *s*, and (much) more so for inversions (figure 3*c*). This contrast to our first model emerges because of the necessity for blue alleles to cross the yellow habitat.

### (c) Theory

Analytically, the situation can be understood as follows. We assume continuous space with diffusion of genes at rate *σ*^2^ and weak selection. Under migration-selection equilibrium for the first patch, the frequency of the blue allele declines approximately exponentially in the adjacent yellow area, with $p(x)\approx \exp\left(-(x\sqrt{2s}/\sigma )\right),$where *x* is the spatial position (electronic supplementary material, information 2.2/[40,41]). Importantly, because *s_e_* ≈ 2*s* in an inversion, the frequency of blue alleles in the standing genetic variation in the yellow habitat, *p*(*x*), will be reduced for an inversion.

When the second blue patch emerges, the frequency of the blue allele will increase from this low starting frequency if its rate of increase, *λ*, is positive. *λ* increases with *s* (relative to dispersal) and with the patch size. Slatkin [41] and Nagylaki [40] found that the critical patch size below which local adaptation is impossible is $L_{crit}=(\pi /2)(\sigma /\sqrt{2s})$: with stronger selection, adaptation is possible in smaller patches. With an inversion, we need to substitute *s* for *s_e_* and the critical patch size is reduced—an inversion can drive parallel adaptation even when unlinked alleles would experience swamping.

In the deterministic situation, the time to establish in the new patch depends on the initial frequency where the new patch emerges (*p*(*x*)) and on the rate of increase, *λ* (electronic supplementary material, information 2.2):

$$T\approx y\frac{\sqrt{2s}}{\sigma }\frac{1}{\lambda }+C,$$

*y* is the distance from the already established patch. The constant *C* has a complicated form, but the time to establishment increases linearly with *y* and is inversely proportional to the rate of increase. Importantly, *λ* increases with *s* faster than $\sqrt{s}$ does (electronic supplementary material, information 2.2); this means that while alleles under stronger selection are initially rarer, this is outbalanced by a more rapid increase when selection changes. Because *s*_e_ > *s*, inversions thus lead to more rapid adaptation in the deterministic model.

However, with realistic population sizes, there will be drift effects. The higher *s*, the more likely it is that the blue allele is not present at all in the standing variation. Waiting for arrival via gene flow can dramatically increase time to adaptation (figure 3*b*). This effect disproportionately affects inversions, as again they experience *s*_e_ ≈ 2*s* rather than *s*.

## 4. Parallel evolution along large-scale environmental gradients

While above we have discussed discrete environments, repeated shallow gradients can also lead to parallel adaptation (e.g. figure 1*c*). Here ‘shallow’ means that the environmental change only impacts fitness on scales that are many times larger than the dispersal distance. Migration between the different gradients must happen via long-distance migration events.

### (a) Case studies

The best-known large-scale inversion clines are in *Drosophila*. Kapun & Flatt [42] have reviewed the evidence for the selective maintenance of inversion polymorphisms in *D. melanogaster*. All of the four ‘common cosmopolitan’ inversions (*In(2 L)t, In(2R)NS, In(3 L)P and In(3R)Payne*: 4.8–17.4 Mb, 962–1900 genes, estimated ages 13 000–180 000 years; [6]) show latitudinal clines that tend to be repeated across continents, with the strongest signals for the *In(3R)Payne* inversion (accounting for about 80% of clinal SNPs in North America; [43]). Clinal variation with altitude, potentially along similar (though steeper) environmental gradients and spatial cline shifts associated potentially with climate change have also been interpreted as support for the role of selection. Inversion clines in *Drosophila* species potentially explain a large proportion of clinal variation in phenotypic traits ([44] and references therein).

Similar clinal inversion patterns to those documented in *D. melanogaster* have been observed in other *Drosophila* species [45,46]. *Drosophila subobscura* has been particularly well studied. There are clines for multiple inversions in its native range in Eurasia that seem to be shifting with changes in climate [47,48]. Latitudinal clines were ‘re-formed’ rapidly in both North and South America, following human-assisted colonization in the 1970s, although with shallower slopes [47].

There are relatively few cases outside *Drosophila* where independent parallel clines have been observed. In the seaweed fly, *Coelopa frigida*, similar clines for chromosome 1 karyotypes (differing by three overlapping inversions, about 10% of the genome, 25 Mb and greater than 2000 genes; [49]) occur on both the east and west coasts of the North Atlantic and must be independently maintained [50]. In the grasshopper, *Trimerotropis pallidipennis,* inversions on four chromosomes show altitudinal clines in multiple, distant transects in Argentina [51]. Finally, common ragweed forms a latitudinal cline in size and phenology in its native continent, North America, and has evolved parallel clines in Europe, Asia and Australia following introduction a few hundred years ago [52]. One major and two minor QTLs underlying flowering time and plant size reside in a putative inversion [52].

### (b) Expectations

We have not modelled the formation of parallel large-scale clines, as this would require an extensive treatment, but we argue that inversions could increase the probability of parallel phenotypic clines. We assume that large-scale clines are maintained by adaptation to a shallow environmental gradient. In *D. melanogaster*, inversion frequencies change by only about 1% per 100 km (based on data in [42]), which is many times the estimated dispersal distance (approx. 1 km per generation [53,54]). In *Coelopa frigida,* the frequency of the *α* arrangement of the chromosome 1 inversion similarly changes from 0.35 to 0.45 over about 300 km [50], but in *Trimerotropis pallidipennis* the frequency gradients are steeper: 0 to 0.8 over about 100 km and 2000 m of altitude [51], probably with shorter dispersal distance than the flies.

**Figure 4.**
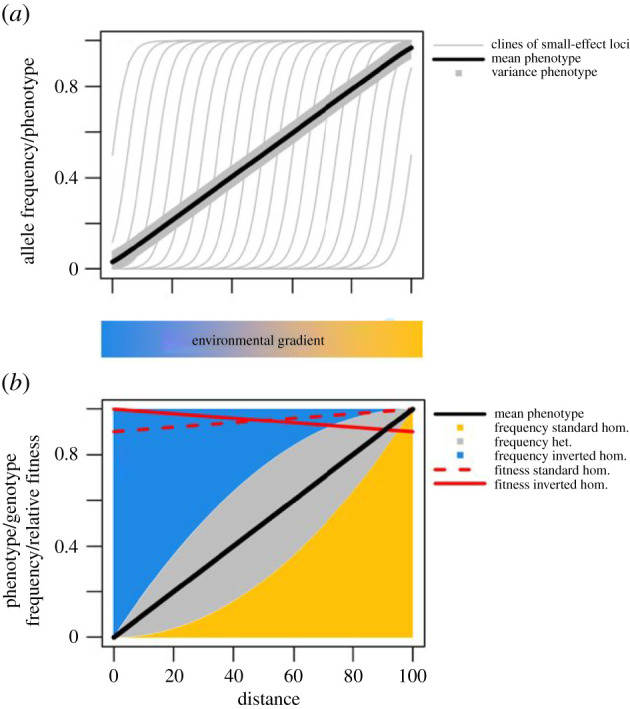
Two models for large-scale clines. In (*a*), the mean phenotype (black line) matches the underlying environmental gradient (blue to yellow) because of staggered clines in multiple loci of small effect (grey lines). The grey box illustrates the constant variance around the mean phenotype. In (*b*), the equilibrium frequency of an inversion (*a*/(*a* + *b*), where *a* is the selection coefficient experienced by one homokaryotype and *b* the selection coefficient experienced by the other homokaryotype), and so the mean of a phenotype influenced by loci within the inversion (black line), matches the environmental gradient because the fitnesses of the two homokaryotypes (relative to the heterokaryotype; for the inverted homokaryotype: 1 − *a*, for the standard homokaryotype: 1 − *b*) vary with the environment. Proportions of the different inversion genotypes are shaded, illustrating the high frequency of maladapted genotypes, even though the mean follows the environmental gradient (e.g. at distance = 50, load = 0.25*a* + 0.25*b*). hom = homokaryotype; het = heterokaryotype.

Polygenic adaptation to shallow environmental gradients, without linkage, is expected to result in a series of staggered clines ([55,56]; figure 4*a*). An inversion bringing together many such loci would be roughly equivalent to one large-effect locus. A simple model then predicts an inversion cline that is steeper than the environmental gradient, centred where the relative fitnesses of the two arrangements are equal. However, this is incompatible with observed patterns. Alternatively, inversion frequencies are maintained by balancing selection at local equilibria that change clinally (figure 4*b*). This model is attractive for stable, large-scale clines because it can combine strong selection with shallow frequency gradients, and is supported by empirical evidence for balancing selection in *Drosophila* and *Coelopa* flies [57–59]. Balancing selection via heterokaryotype advantage is expected under models of the accumulation of deleterious recessive alleles within inversions [60] (Also see Berdan *et al*., [61]). However, this model implies substantial load due to the high frequencies of locally maladapted inversion genotypes at most points in the cline (figure 4*b*). Alternatively, inversion polymorphism may be maintained by frequency-dependent selection in heterogeneous environments, as argued by Fuller *et al*. [62] for *D. pseudoobscura*, with large-scale clines generated by change in the mix of habitats.

What is the probability that colonization of a new environmental gradient will generate a parallel phenotypic cline under each of these alternative models: polygenic adaptation with staggered clines versus an inversion cline maintained by local balancing selection? Under the polygenic model, spacing of clines (figure 4*a*) means that only a small proportion of adaptive loci are expected to be polymorphic in a group of colonists from any single location, even if the number of colonists is large. This limited variation would allow adaptation only to a narrow environmental range. By contrast, an inversion under balancing selection is expected to be polymorphic over a wide range in the source region, so that variation to re-establish a full cline is present even in a small founding group. Therefore, we might expect inversions to disproportionately contribute to parallel large-scale clines.

We have assumed that under the polygenic model, there is no redundancy in genetic architectures and most loci contributing to adaptation are fixed in any given location. However, under highly polygenic adaptation, there are often multiple ways to reach the same phenotypic outcome and local additive genetic variation (*V*_A_) is large. In this case, even a small number of colonization events might introduce sufficient variation to generate a large-scale cline, and the difference between inversions and collinear architectures might be less pronounced than discussed above.

## 5. Factors that facilitate or hinder a role of inversions in parallel evolution

### (a) The strength of selection and the number of adaptive loci in the inversion

As illustrated by figures 2 and 3, the strength of selection is crucial to determine the advantage (or disadvantage) of an inversion over collinear architectures in parallel evolution. Under some circumstances, by leading to higher effective selection, inversions strongly increase the equilibrium frequency of the adaptive alleles (i.e. lead to better adaptation), speed up the adaptive process, or enable parallel adaptation where it would not be possible with a collinear architecture. The exact range of selection coefficients where this is true depends on the spatial model and other parameters (e.g. dispersal rate).

In our models, we have considered only two adaptive loci, but inversions can contain adaptive alleles at numerous loci and contribute to multiple traits (e.g. [5,9]). The larger the number of alleles with positive effects, the greater will be the difference between *s* and *s*_e_. We thus expect that, under constant *s*, the differences in figures 2 and 3 would become (much) more pronounced with large numbers of adaptive alleles. On the other hand, an arrangement might also contain locally or globally deleterious alleles, which are harder to remove than in collinear regions due to reduced recombination [14,60]. Such alleles can reduce the advantage the inversion experiences in some scenarios.

### (b) Dispersal patterns

In many taxa, dispersal includes both short-range dispersal and rare long-range migration events. In our first model, parallel evolution is driven by the latter, in our second model by the former. In the second model, inversions suffer from a disadvantage because they experience stronger selection when crossing the ‘wrong’ habitat, thus needing more time before they can contribute to parallel evolution. In the first model, where long-range migration between patches of the same habitat is possible, there is no such disadvantage, and inversions lead to more rapid adaptation. It thus seems likely that inversions play a larger role when similar habitats are close together (relative to dispersal rates) or when long-range dispersal is possible. Our empirical examples show that inversions sometimes do seem to play a role in rapid adaptation across large distances in systems where long-range migration seems unlikely (e.g. sticklebacks); this might be due to factors such as balancing selection that modify the predicted outcome (see below).

### (c) The patch size

Parallel evolution can become impossible when a habitat patch is too small for selection to overcome the inflow of maladapted alleles (§3). The critical patch size is smaller with an inversion because of the effectively stronger selection. Even with larger patches, local adaptation will be less pronounced with collinear architectures unless selection is very strong. Only when the patch is very large relative to dispersal does the extent of adaptation away from the patch boundaries become independent of the genetic architecture. We thus expect inversions to contribute particularly to parallel adaptation where the environment varies on very small scales. For example, in *L. saxatilis*, very small areas of Crab habitat can interrupt a cliff [36]; it would be interesting to test whether inversions contribute disproportionately to adaptation in such areas.

### (d) Soft versus hard selection

Hard selection occurs when the population density varies in response to selection. In our examples we have only modelled soft selection. We expect that hard selection could substantially alter expectations in favour of inversions. For example, as we have seen, adaptation in small patches is threatened by swamping and might only be possible with an inversion. We expect the parameter range where this is the case to be larger under hard selection: with hard selection, the population size in a small patch will be reduced due to a high frequency of maladapted alleles, which in turn increases the inflow of maladapted alleles from the surrounding habitat, making stronger selection necessary to maintain high levels of local adaptation [63].

### (e) The extent of environmental parallelism

Most models of parallel evolution (including ours) assume that the environments are perfectly parallel, i.e. that selection coefficients are the same in all blue and in all yellow patches. However, in nature this may not be the case (e.g. in stickleback lake-stream divergence [64]), so that different locations do not require the same set of adaptive alleles. While inversions might be particularly useful to transport a set of defined alleles between locations, they are less flexible for local adjustments. For example, if adaptation in the first blue patch involves large body size and red colouration, and alleles for both are in the same inversion arrangement, then parallel adaptation using the same inversion is difficult if large body size, but green colouration is favoured in the second blue patch. This is because alleles co-located in an inversion are hard to decouple (however, this effect is weakened by gene flux between arrangements e.g. via double cross-over, particularly in large inversions; [65]). We could thus expect inversions to contribute less to parallel adaptation where the environment expresses many non-parallel features, at least in the short term. Over longer timescales, inversions could accumulate location-specific adaptive mutations.

An important message is that because different copies of the same arrangement can vary in content, the repeated use of the same inversion does not necessarily mean that the actual genetic basis of adaptation is the same. If inversions are old and geographically widespread, as has been shown in some cases (examples above, [66]), it is likely that such within-arrangement diversity has evolved. An example for within-supergene evolution after its initial formation is described for *Heliconius* butterflies in this issue [67]. Studying inversion content is a key challenge for the future of research on inversions involved in adaptation, complicated by the high linkage disequilibrium that makes studying the effects of individual loci more difficult.

### (f) Balancing selection

Evidence for balancing selection is commonly reported for inversion polymorphisms [6]. Most commonly, it is attributed either to overdominance or to spatially and/or temporally-variable selection (table 1 in [6]). Inversions remaining polymorphic within populations for a long time may accumulate deleterious recessive alleles and so tend to become overdominant [60].

Balancing selection on inversions that maintains polymorphism in both alternative habitats is likely to increase their role in parallel adaptation. In particular, it will aid or even be a necessary requirement for the crossing of alternative habitats (figure 1*b*, figure 3*c*). It could also contribute in scenarios not discussed here, for example a version of figure 1*a* without gene flow between islands, where adaptation with a shared genetic basis is only possible if a polymorphism is maintained in the standing genetic variation on both islands. Maintenance of both alleles in both habitats would increase the chances of the adaptive arrangement being available in the newly blue deme on island 2, and this may apply to the *Timema* example [18]. Both of these effects may contribute to parallelism in *Littorina* [36]. Finally, parallel large-scale inversion clines (figure 1*c*) are more likely to result from colonization if partly maintained by balancing selection, as argued above. The seaweed fly, *Coelopa frigida*, provides a particularly clear example of parallel clinal variation in the presence of heterosis [50]. However, the empirical question remains whether balancing selection is more common on inversions than on other genomic regions.

## 6. Avenues for future research

We have highlighted empirical and conceptual support for a role of inversions in parallel evolution. However, the number of empirical systems where both parallel evolution has been found and a role of inversions has been tested is small and comes from a limited range of taxa. In particular, work on plants is rare. On the other hand, if inversions are found to contribute, their importance may sometimes be overestimated because of the strikingly large blocks of outliers they can form in the genome. The *relative* contribution of inversions compared to collinear architectures is rarely known. A research programme addressing the importance of inversions in parallel evolution needs to combine a range of approaches:

1. Test for parallel evolution: It is important to distinguish repeated phenotypic patterns caused by repeated colonisation events from the same origin from true parallel evolution (i.e. repeated phenotypic divergence associated with repeated demographic divergence processes; [39,68]). This might be achieved using demographic modelling of the population histories.
2. Analyses of the genomic basis of local adaptation in each location: Typically, outlier scans for selection (e.g. *F*_ST_ scans) are used, but due to the high probability of false positives additional analyses (e.g. QTL mapping for divergent traits, hybrid zone analysis) are helpful [69].
3. Identifying inversions in each location: in each location, it needs to be tested whether polymorphic inversions occur and whether they show elevated differentiation. In outlier scans, inversions containing loci under divergent selection can appear as large blocks of outliers because of the strong LD among SNPs. However, searching for blocks of outliers is not sufficient: first, inversion polymorphisms not contributing to divergence cannot be detected this way. Second, other clusters of loci contributing to local adaptation ('genomic islands of divergence'), especially in low-recombination regions, can also form high-differentiation blocks. Third, *F*_ST_ scans do not provide arrangement frequencies and thus do not allow for formal tests for elevated differentiation. It is thus necessary to use independent methods for identifying inversions and determining arrangement frequencies in each population. These include methods based on linkage disequilibrium [21,70] and read-based methods e.g. testing for unusual relative positions of paired-end reads [71]. With long-read sequencing data the reliability of these methods increases.
4. Testing the relative contribution of inversions to parallel evolution: maybe the simplest test for a disproportionate contribution of inversions to parallel evolution is to extend an analysis of outlier sharing to inversions. For example, analyses of parallel evolution based on SNPs or genomic windows often test to what extent outlier sets overlap between different geographical locations (e.g. [23,72,73]). One could ask whether inversions are shared more extensively between locations than outliers outside inversions. However, traits rarely evolve fully in parallel. To test for the relative contribution of inversions (and other loci) to parallel evolution, one would specifically need to quantify the contribution of these genomic regions to the parallel between-population variation in any trait that shows parallel evolution. To our knowledge, such a framework for studying parallel evolution is not established yet and will be an important contribution to our understanding of the genomic architectures underlying parallel evolution.

Future theoretical work should explore the interactions between different factors hindering and facilitating a role of inversions in parallel evolution and explore further historical models and a wider range of parameters (including polygenic selection) than we could consider here. Theoretical and empirical work to separate the evolution of inversions as vehicles for locally adaptive alleles from the evolution of their content will also be crucial.

## Data Availability

Code for the simulations (R scripts) and the analytical part (Mathematica notebook) is available in the electronic supplementary material, information 1 and 2 [74].
